# Impact of protein–chromophore interaction on the retinal excited state and photocycle of *Gloeobacter* rhodopsin: role of conserved tryptophan residues†

**DOI:** 10.1039/d3sc02961a

**Published:** 2023-09-06

**Authors:** Ramprasad Misra, Ishita Das, András Dér, Gábor Steinbach, Jin-gon Shim, Wayne Busse, Kwang-Hwan Jung, László Zimányi, Mordechai Sheves

**Affiliations:** a Department of Molecular Chemistry and Materials Science, Weizmann Institute of Science Rehovot 76100 Israel mudi.sheves@weizmann.ac.il; b Institute of Biophysics, Biological Research Centre, Eötvös Loránd Research Network Szeged H-6726 Hungary zimanyi.laszlo@brc.hu; c Cellular Imaging Laboratory, Biological Research Centre, Eötvös Loránd Research Network Szeged H-6726 Hungary; d Department of Life Science and Institute of Biological Interfaces, Sogang University Seoul 04107 South Korea; e Institute for Biology, Experimental Biophysics, Humboldt-Universität zu Berlin Berlin 10115 Germany

## Abstract

The function of microbial as well as mammalian retinal proteins (*aka* rhodopsins) is associated with a photocycle initiated by light excitation of the retinal chromophore of the protein, covalently bound through a protonated Schiff base linkage. Although electrostatics controls chemical reactions of many organic molecules, attempt to understand its role in controlling excited state reactivity of rhodopsins and, thereby, their photocycle is scarce. Here, we investigate the effect of highly conserved tryptophan residues, between which the all-*trans* retinal chromophore of the protein is sandwiched in microbial rhodopsins, on the charge distribution along the retinal excited state, quantum yield and nature of the light-induced photocycle and absorption properties of *Gloeobacter* rhodopsin (GR). Replacement of these tryptophan residues by non-aromatic leucine (W222L and W122L) or phenylalanine (W222F) does not significantly affect the absorption maximum of the protein, while all the mutants showed higher sensitivity to photobleaching, compared to wild-type GR. Flash photolysis studies revealed lower quantum yield of *trans*–*cis* photoisomerization in W222L as well as W222F mutants relative to wild-type. The photocycle kinetics are also controlled by these tryptophan residues, resulting in altered accumulation and lifetime of the intermediates in the W222L and W222F mutants. We propose that protein–retinal interactions facilitated by conserved tryptophan residues are crucial for achieving high quantum yield of the light-induced retinal isomerization, and affect the thermal retinal re-isomerization to the resting state.

## Abstract 2

The conserved tryptophan residues in the vicinity of the retinal in microbial rhodopsins affect the retinal excited state charge distribution, increase the isomerization quantum yield, and affect the retinal double bond reisomerization rate during photocycle.

## Graphical abstract

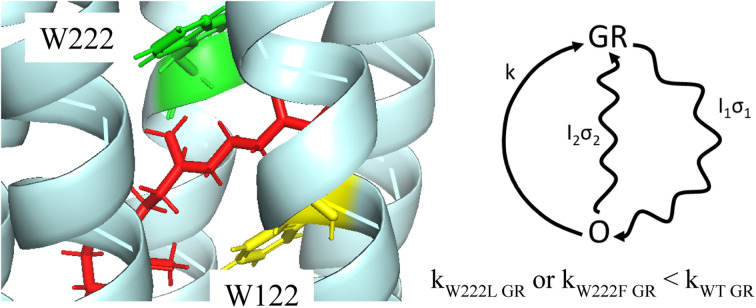

## Introduction

**Fig. 1:**
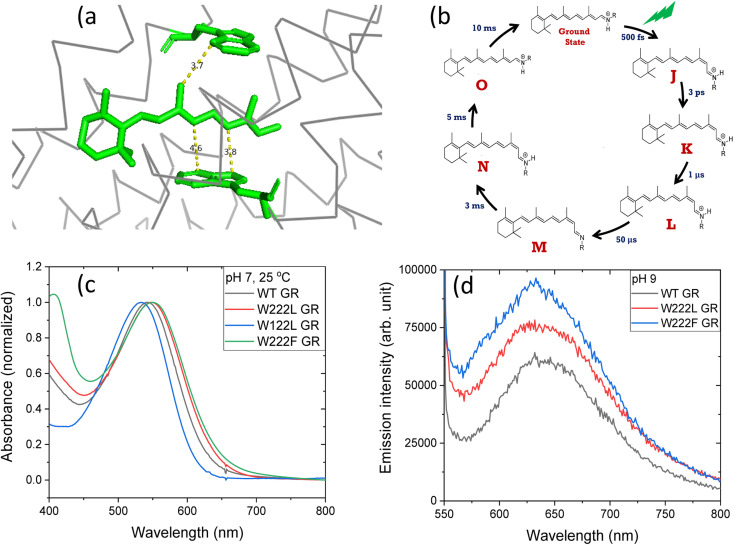
(a) The position of tryptophan residues 222 (above) and 122 (below) with respect to the retinal chromophore, as obtained from the crystal structure of GR (PDB ID: 6nwd). The distances reported are in Å. (b) A typical photocycle scheme of microbial rhodopsins, consisting of spectroscopically distinct photo-intermediates denoted as J, K, L, M, N and O. (c) Normalized absorption spectra of GR and its W222L, W122L and W222F mutants at 25 °C and pH 7. (d) Recorded emission spectra (arb. unit) of similar concentrations of GR and W222L GR and W222F GR mutants at 25 °C and pH 9 (50 mM Tris buffer), obtained by exciting the corresponding samples at 540 nm.

Retinal proteins serve as photo-receptors, and are responsible for vision in animals and perform several functions, *e.g.*, proton and ion pumping, ion channel and ion sensor in lower organisms like bacteria and archaea.^1–3^ In microbial rhodopsins the covalently bound all-*trans* retinal chromophore is sandwiched between at least two tryptophan (Trp/W) residues (Fig. 1a).^1,4^ Light excitation of their retinal protonated Schiff base (RPSB) leads to isomerization to the 13-*cis* isomer which thermally re-isomerizes to the all-*trans* configuration following a cascade of processes which leads back to the initial protein structure. This photocycle consists of spectroscopically distinguishable intermediates, identified by J, K, L, M, N and O (Fig. 1b).^1,5^ The quantum yield (*ϕ*) of the *trans*–*cis* isomerization of RPSB in microbial rhodopsins is very high and specific to the C_13_=C_14_ double bond, although the other double bonds of the RPSB chromophore can isomerize following irradiation in solution (without the protein) or in protein under special conditions.^6,7^ The quantum yield of retinal isomerization of bacteriorhodopsin (BR), the most studied microbial rhodopsin, for example, is 0.6.^8^ Following light absorption an unusually strong dipole is induced in the retinal polyene, and the interaction between the retinal and surrounding tryptophan residues was proposed to stabilize the large induced dipole developed in the retinal excited state in BR.^9,10^ Furthermore, it was proposed that Trp residues in the vicinity of the retinal respond to the retinal excited state formation and change their absorption.^11^ It was further reported that charge redistribution along the retinal polyene occurs within 200 fs following photon absorption. Coupling between BR RPSB and the protein moiety, especially the steric repulsion between the indole ring of Trp182 and the retinal was reported.^12–14^ Upon photoactivation, the initially twisted chromophore of BR displaces the conserved Trp182 of helix F on the cytoplasmic side of the protein, while dislocating a crucial water molecule on the extracellular side. During M → N transition in the BR photocycle the environment surrounding Trp182 changes from hydrophobic to hydrophilic, as was shown using ultraviolet resonance Raman (UVRR) spectroscopy.^15^ In the N-intermediate the site of Trp182 in the protein binding pocket may be accessible to the water molecules. Atomic contacts between the bulky side chain of Trp215 and the C_20_ methyl group of the retinal chromophore promote relaxation of the RPSB from the 13-*cis* to the all-*trans* form in the light-driven outward Na^+^ pump *Krokinobacter* rhodopsin 2 (KR2).^16^ Time-resolved UVRR spectroscopy revealed that the environment around Trp215 becomes less hydrophobic at 1 ms after photoirradiation, and recovers to the unphotolyzed state with a time constant of about 10 ms.^17^ Recently, time-resolved absorption as well as Raman spectroscopic studies were employed to investigate the effect of ultrafast electric field change on the photocycle of BR. The change in the aromatic residues induced by the high dipole moment of the retinal chromophore in BR was also observed in recent deep-UV femtosecond stimulated Raman spectroscopy.^18^

The crystal structure of *Gloeobacter* rhodopsin (GR)^4^ revealed that Trp222 and Trp122 residues reside near the 9-methyl group and between the 10th and 12th position of the retinal chromophore, respectively (Fig. 1a), which are equivalent to Trp182 and Trp86 residues in BR.^19^ Several studies reported the effect of these conserved tryptophan residues on the absorption, photophysical and photochemical processes of the different retinal proteins.^20–23^ Replacement of bulky tryptophan residues by phenylalanine or by non-aromatic and smaller leucine is expected to alter the electronic and steric environment around the retinal, and thereby may affect the charge distribution along the retinal excited state. The effect of electric field change around the retinal chromophore on the excited state reactivity of retinal proteins and the protein photocycle was previously suggested.^9,24–26^ Despite crucial roles played by the conserved Trp residues,^27–30^ their effects on the retinal isomerization quantum yield and on the photocycle of microbial rhodopsins are not yet well understood.

Here we report the effect of the change in the protein electrostatic environment on GR's RPSB photocycle through mutation of the highly conserved Trp residues at 222 and 122 positions around the retinal chromophore. Strikingly, it was observed that the retinal isomerization quantum yield is significantly lower in the W222L as well as W222F mutants than in WT GR, suggesting that the interaction between the retinal and surrounding Trp residues is crucial in controlling the high light-induced dipole in the retinal excited state, and controlling the charge redistribution along the retinal polyene during the excited state lifetime. The replacement of the aromatic W222 with leucine affects the absorption spectrum of the first (observable) ground state intermediate, K. It also accelerates the deprotonation of the RPSB and decelerates the recovery of the resting state presumably by hindering retinal re-isomerization.

## Materials and methods

Wild-type GR (WT GR) and its W222L, W222F and W122L mutants were expressed as recombinant proteins in Luria–Bertani (LB) liquid medium in *Escherichia coli*, and were purified following the procedure reported earlier^31^ (see the ESI† for details). As the GR mutants are highly sensitive toward light, the purification protocol was performed at 4 °C in the dark. The steady-state absorption measurements (using an Agilent 8453 diode-array spectrophotometer from Agilent Technologies, Palo Alto, CA) were carried out in 50 mM buffer solutions of pH 7 (phosphate) and 9 (carbonate), also containing 300 mM NaCl. The emission spectra were recorded using a fluorimeter from Jobin Yvon Horiba. The continuous irradiation was done using a Schott 250 W cold light source (Carl Zeiss Microscopy, Jena, Germany), which is equipped with a heat-absorbing filter and an optic fiber (level 4B). The light intensity and time of irradiation were optimized for all the samples in such a way that the light effect does not saturate at the given light intensity and exposure time. For flash photolysis measurements the samples were prepared in 50 mM buffer (50 mM KH_2_PO_4_, 50 mM CHES) of pH 9 with 100 mM NaCl as reported by Miranda *et al.*^32^ The time-resolved experiments were done in 1 cm (measuring light) × 4 mm (laser excitation) quartz cuvettes, with opaque walls facing the laser beam in order to minimize polarization effects. The laser (Nd-YAG 2nd harmonic, 532 nm, 7 ns, Continuum Surelite II) was vertically polarized. Multichannel measurements were performed by gating the CCD detector (Andor iStar), mounted on a Horiba Jobin Yvon spectrograph, at logarithmically quasi-equidistant time delays after the laser pulse, with gradually increasing, appropriate gate pulse widths. The white measuring light (tungsten halogen light bulb) was chopped before the sample to minimize actinic effects, the open time of the shutter was 20 millisecond (ms), and CCD detector gating took place a few ms after the shutter opening. Single wavelength kinetics experiments were done in the same setup, at 620 nm, with the continuous measuring light detected by a photomultiplier, using a high pass (>600 nm) filter before the sample, again, with the intention to decrease the actinic effect of the measuring light. The fixing of the GR W222F sample in acrylamide gel slabs was done using the protocol reported elsewhere.^32^

## Results and discussion

### Absorption and continuous irradiation of GR and its W222L and W122L mutants

**Fig. 2:**
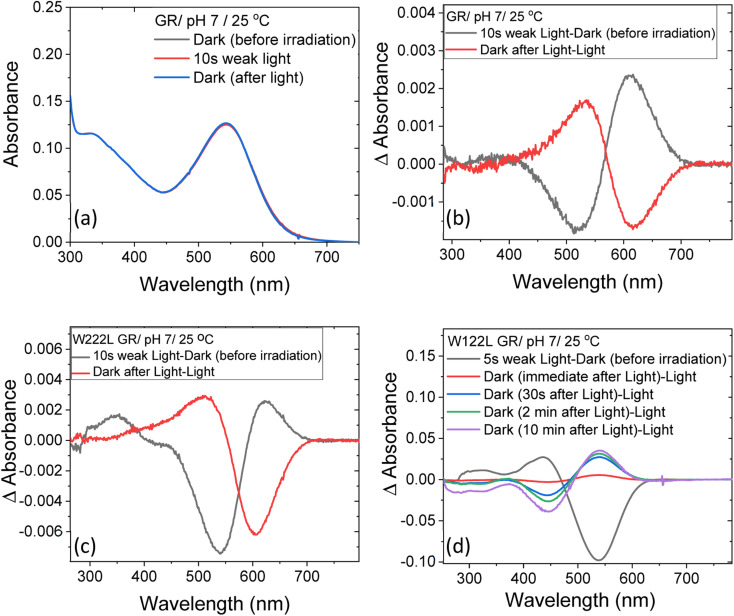
The change in steady-state absorption of (a) GR due to irradiation with <500 nm cut-off filter at pH 7. The spectra recorded before, during and after illumination was terminated are presented. (b) The difference spectra obtained by subtracting consecutive spectra recorded during irradiation as indicated. The difference spectra for (c) W222L and (d) W122L mutants, obtained by the procedure described in (b). The corresponding absorption spectra of these mutants before, during and after irradiation are reported in Fig. S3.†

At neutral pH and room temperature (25 °C), wild-type GR and its W222L and W122L mutants showed absorption band with maxima (*λ*_max_) at 542 nm, 548 nm and 533 nm, respectively (Fig. 1). As the titration of GR and W222L GR revealed that the p*K*_a_ of their primary counter-ion is 4.4 and 6.5 (Fig. S1†), respectively, to avoid the effect of counter-ion protonation on the protein photocycle, the kinetic absorption studies were performed at pH 9 (see below). The *λ*_max_ of GR, W222L GR and W122L GR at pH 9 are 540, 541and 533 nm (Fig. S2†), respectively, indicating that the Trp mutations do not significantly affect the absorption spectra of GR. We have irradiated the GR using white light with a >500 nm filter, the intensity of which was below the saturation level. Irradiation of GR at pH 7 yielded a photoproduct at about 615 nm which thermally decayed back to the original pigment (Fig. 2 and S3†). The W222L GR mutant showed more product formation than GR upon illumination under similar conditions. The difference spectrum showed increase in intensity at 622 nm and 350 nm with decrease in intensity at 540 nm due to irradiation with white light with a <500 nm cut-off filter. The band at 350 nm indicated that a part of the protein was bleached due to irradiation.^33^ Following the irradiation, the red-shifted photo-product thermally decayed to the original pigment. At pH 9, both proteins yielded more photoproducts relative to pH 7 (Fig. S4†). Native GR showed similar behavior to that at pH 7 but also yielded a denatured product as was evident by the appearance of an additional band at 364 nm. The W222L mutant showed changes in intensity more than twice due to irradiation at pH 9 than at pH 7. Unlike wild-type GR and the W222L mutant, the W122L GR mutant showed unusual instability to irradiation with similar intensity. White light irradiation at pH 7 led to significant decrease of the protein chromophore band with appearance of two bands with maxima at 454 and 338 nm, respectively (Fig. 2). A fraction of the protein pigment band thermally reformed in the dark as the 454 nm absorbing species reconverted to the original pigment. Irradiation of W122L GR protein with white light for 5 seconds at pH 9 led to complete pigment denaturation as evident by formation of a band at 361 nm (Fig. S4†). It was previously suggested that the rate-controlling step in retinal protein denaturation is the hydrolysis of the protonated Schiff base bond.^33^ Therefore, it is possible that substitution of the Trp residue by the smaller leucine residue increases the accessibility of water molecules (following irradiation), facilitating the protonated Schiff base hydrolysis.

### Photocycle of GR and its W222L and W222F mutants

**Fig. 3:**
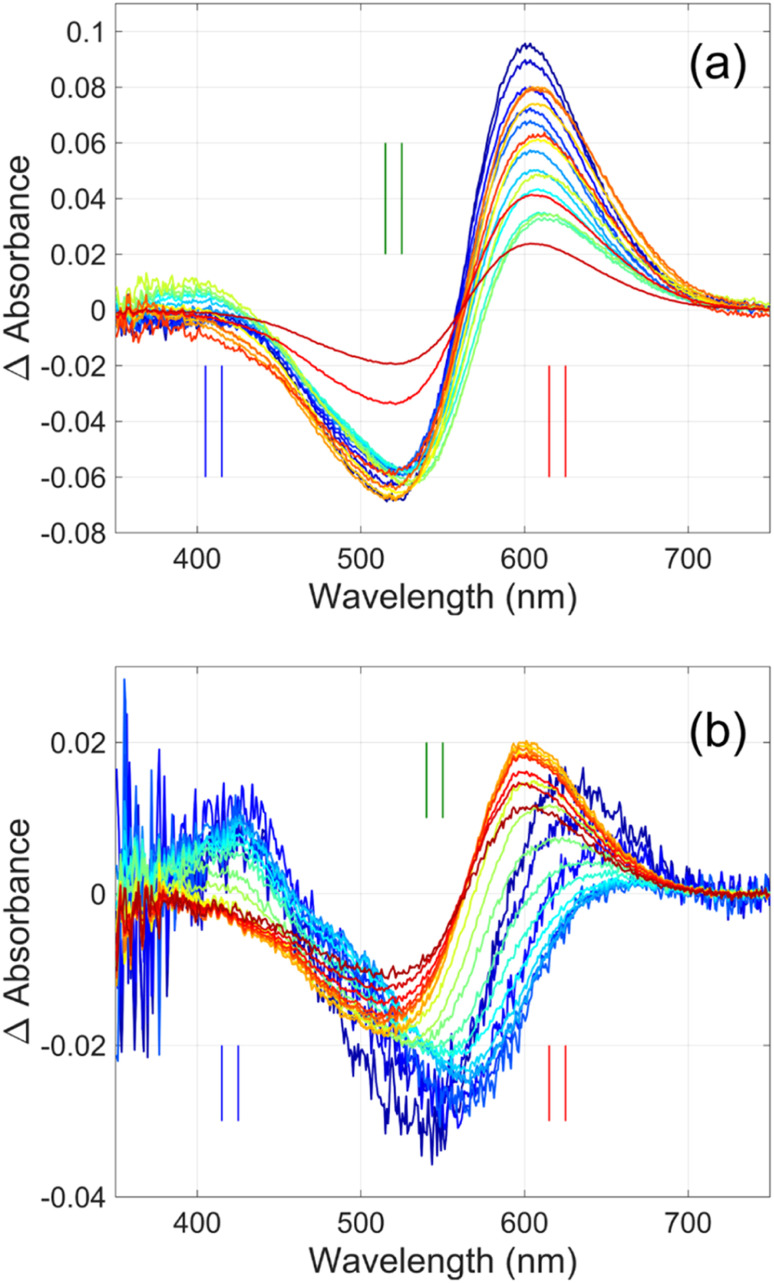
Time-resolved difference spectra of (a) GR and its (b) W222L mutant. The rainbow color code corresponds to the delay times as in Fig. 4(a) and (b). The amplitudes of the spectra were corrected for bleaching. Vertical lines mark the regions for averaging the spectra in order to obtain “single wavelength” kinetics in Fig. 4(a) and (b). The spectra are SVD reconstituted for noise filtering, using the 4 most significant SVD components.

**Fig. 4:**
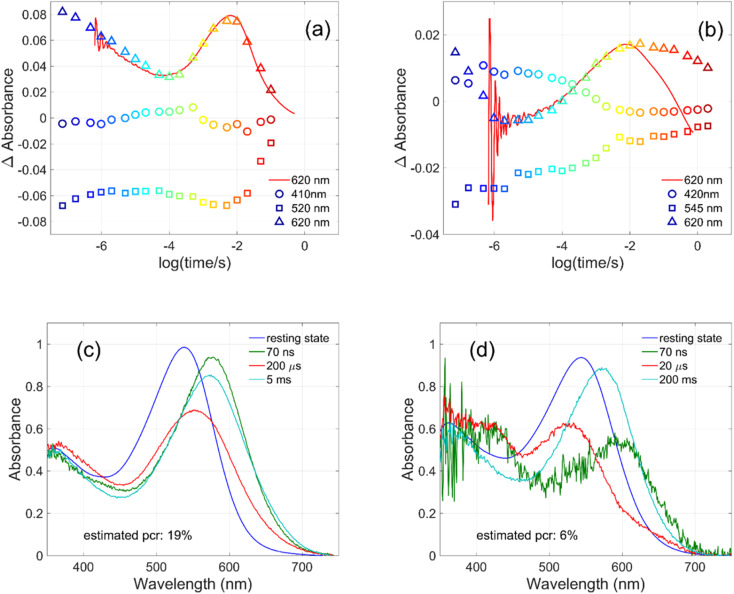
Kinetics measured for (a) GR at 410, 520 and 620 nm and for (b) W222L GR at 420, 545 and 620 nm (symbols), and directly measured at 620 nm (red lines). Possible intermediate mixture spectra of (c) GR at 70 ns, 200 μs and 5 ms and of (d) W222L GR at 70 ns, 20 μs and 200 ms, by assuming the indicated photocycling ratios.

Flash photolysis experiments were carried out at 20 °C (controlled). The time-resolved difference spectra of WT GR and W222L mutant are shown in Fig. 3. The W122L mutant proved to be highly light sensitive and bleached completely during 10 laser flashes, therefore, no results could be obtained on this mutant. For WT GR, early difference spectra were taken by averaging 50 flashes, a number that was gradually decreased later to 10. For the W222L mutant, 10 flashes were averaged at each delay time. Also, 10 flashes were averaged for both samples during the 620 nm single-wavelength kinetics experiments. Even after correction for the absorption difference at the laser wavelength (532 nm), the photocycling ratio (pcr) and, therefore, the quantum yield is much smaller in the W222L mutant than in WT GR. The values of pcr (0 < pcr < 1) were obtained by adding to the difference spectra (Fig. 3) the appropriate pcr-multiplied spectra of the resting state (Fig. 4c and d; blue curves) and normalizing by pcr:*A*_intermediate_ = (*D*_intermediate_ + pcr × *A*_resting state_)/pcr

The criteria for the resulting intermediate (or intermediate mixture) absolute spectra (Fig. 4c and d) and, in particular, for a reasonable O-intermediate spectrum (cyan curves in Fig. 4c and d) close to the end of the photocycle, but before the start of the recovery of the initial form, were non-negativity and acceptable shape (*i.e.*, single band and usual width for retinal proteins). Hence, the quantum yield (QE) of the W222L mutant photocycle relative to that of WT GR is obtained as (pcr_W222L_/pcr_WT_)/(*A*_W222L, 532_/*A*_WT, 532_) = (6/19)/0.93 = 0.34.

In addition, their photocycle kinetics and intermediate spectra are rather different. Although the resting state absorption spectra of the WT and the W222L mutant are very similar, the K-intermediate of the mutant is substantially red-shifted compared to WT and, judged by the corresponding early absolute spectrum, it may be in rapid equilibrium with a blue shifted form. The decay of the K-state and the deprotonation of the RPSB are much faster in the mutant, resulting in more M-intermediate accumulation apparently in rapid equilibrium with L and/or N-intermediates. This finding also correlates with the higher p*K*_a_ of the counterion in the mutant (Fig. S1†), indicating a higher proton affinity of the extracellular side neighborhood of the RPSB. The decay of the O-intermediate, presumably the re-isomerization of the retinal, is very much hindered in the mutant. This step could also be due to the internal proton transfer from the primary acceptor to the proton release site (Asp115),^4^ or directly to the extracellular solvent, but the re-isomerization is a likely candidate. We propose that the continuous red measuring light in the single-wavelength experiment drives this step, whereas the very short exposure to the (white, but it should be only the red component that matters) measuring light in the multichannel experiment has a more negligible actinic effect. This may be the reason for the (reproducible) discrepancy seen in Fig. 4b. In WT GR thermal re-isomerization, *i.e.*, decay of the O-intermediate, is sufficiently fast and, therefore, the actinic effect of the 620 nm measuring light is not detected. The photocycle of WT GR, its kinetics and estimated intermediate spectra resemble by and large those reported earlier.^18^ The authors claimed that the O-intermediate is twisted all-*trans*, while the N-intermediate is still 13-*cis*. The difference we observe in the kinetics and consequent light sensitivity of the O-intermediate between WT and W222L GR could be caused by the O-state of the mutant having the 13-*cis* configuration.

The most striking observation is associated with the decreased quantum yield of the photocycle formation in the W222L mutant relative to the wild type. The retinal isomerization is very specific to one double bond (C_13_=C_14_), and its quantum yield in retinal proteins is very high.^34^ For BR, the isomerization quantum yield in the excited state was measured as 0.6 while the remaining fraction decays to the ground state.^6^ The question arises which factors control the high quantum yield and the specificity of the double-bond isomerization. All retinal proteins are characterized by conserved aromatic residues in the vicinity of the retinal chromophore, while in bacterial rhodopsin one tryptophan is located next to the C_13_=C_14_ bond (W122 in GR), and in GR and BR for example a second tryptophan is located next to Me-9, close to the β-ionone ring.^4,21^ The light-induced dipole was measured in BR by second harmonic generation (SHG), two-photon absorption, optical rectification and terahertz radiation studies. It was demonstrated that indeed a large light-induced dipole is developed in bacteriorhodopsin following light absorption,^9,10,35–37^ and was found to be about double that of free RPSB in solution (without the protein). The optical rectification studies estimated that the projection of the induced dipole on the membrane normal is 11 D, corresponding to the displacement of a full charge over approximately half the length of the retinal chromophore.^10^ It was proposed that the large induced dipole is enhanced in BR by retinal–protein interaction in the vicinity of the β-ionone ring, which might be attributed to interaction with Trp residues.^9^ Furthermore, it was proposed that the Trp residues respond to the retinal induced dipole, and change their absorption during the excited state lifetime.^11^ We propose that the Trp residues not only facilitate the large induced dipole formation in the retinal excited state in retinal proteins but also assist in directing the redistribution of charge during the excited state lifetime in such a way that most of the positive charge is tunneled to the C_13_=C_14_ bond to allow efficient isomerization with high quantum yield. Therefore, once Trp222 is mutated in GR, the quantum yield is significantly decreased.

The effect of the mutation of the conserved Trp residues with phenylalanine (F) has been studied on the photocycle of BR and other proteins.^38,39^ The proton pumping activity of GR W222F mutant is about 9% of that of WT GR.^39^ It was reported that during the photocycle of W222F GR, the formation of the L-intermediate follows the all-*trans* to 13-*cis* photoisomerization but does not lead to deprotonation of the chromophore.^39^ These results indicate that the interaction between the protein moiety and the RPSB is crucial for facilitating the proton transfer process. It was proposed^38,39^ that the mutation affects the photocycle due to steric effect and modified retinal–protein steric interactions.

**Fig. 5:**
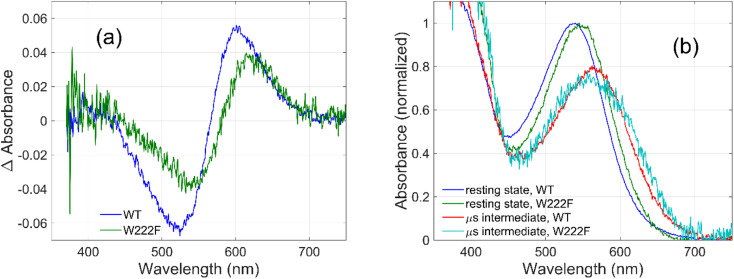
Difference spectra (a) measured on GR and on the W222F mutant at 3 μs after laser flash excitation, representing the first (K) intermediate. Corresponding normalized absolute spectra (b) of the resting state and the K intermediate obtained by adding the appropriately scaled resting state absolute spectra to the difference spectra in (a).

To evaluate whether the quantum yield of the photocycle will also be affected by the substitution of Trp to a modified aromatic core, we have prepared the W222F mutant of GR and studied its photocycle. The absorption maxima of the W222F mutant at pH 7 and 9 are 550 and 545 nm, respectively (Fig. 1c and S2†). As the photocycle of the W222F GR mutant has recently been thoroughly studied,^39^ here we summarize our important relevant findings only. Fig. 5a shows the difference spectrum measured in the μs range after photoexcitation of GR and the W222F mutant, characteristic of the first, K intermediate. The signal amplitude for the mutant is smaller, reflecting a somewhat smaller quantum efficiency. Fig. 5b shows the normalized absolute spectra of the resting state, as well as the calculated μs intermediate spectra with estimated pcr values (see the description above for W222L) of 0.19 (WT) and 0.14 (W222F). If we also take into account that the extinction of W222F at the 532 nm laser wavelength is 97% of that of WT, then the estimated quantum efficiency for W222F is 75% of that of WT. Therefore, modification of Trp to phenylalanine still reduces the isomerization QE but not as much as modification to leucine in which the QE was estimated as 34% of that of WT (described above). We propose that the aromatic core of phenylalanine can still interact with the retinal chromophore while this interaction is diminished in the leucine mutant. The space occupied by Phe and Leu is similar in size although different in shape, and both are less bulky than Trp, but Phe, like Trp, is an aromatic residue. Therefore, it is expected that with Leu in position 222 both the steric and the electronic effects of the native Trp on the retinal excited state and isomerization are altered, whereas with Phe mostly the steric and to a lesser degree the electronic effects.

The high quantum yield in WT GR, and the double-bond isomerization specificity are probably achieved by adequate charge redistribution along the retinal which is controlled by the interactions with the protein Trp residues in the vicinity of the retinal polyene, and possibly with negatively charged residues in the vicinity of the protonated Schiff base linkage. The intact structure of the retinal chromophore may also contribute to adequate charge redistribution. Previous work with free retinal analogs in solution (without the protein) demonstrated that the isomerization quantum yield was affected by retinal modifications.^40^ In addition, QM/MM models were used to show that electron donating or withdrawing substituents of the retinal chromophore caused a decrease or an increase in the electronic mixing of the first two excited states which, in turn, affects the photoisomerization rate.^41^ We note that the steric retinal–protein interactions may contribute as well to the specificity of the double bond isomerization, and can affect the photocycle and the biological activity as was previously suggested.^35^ Since the Trp222 mutation affected the quantum yield and the charge redistribution in the retinal excited state, it is possible that the lifetime of the excited state and the emission intensity will be affected as well.^41,42^ Therefore, we have monitored and compared the emission spectra of GR and the W222L and W222F GR mutants of same concentration (Fig. 1d). The emission maximum of WT GR at pH 9 was found to be at 639 nm, while that of W222L GR and W222F GR are at 636 and 632 nm, respectively. The emission intensities of the W222L and W222F GR mutants were significantly higher than that of wild-type indicating that the excited state is affected by the mutations. However, the emission intensity did not follow the QE order since the intensity of W222F is higher than that of W222L although the latter has a lower QE. It is possible that the excited state lifetime and the emission intensity are affected by more factors as was previously suggested.^43^

### Photostationary states and photocycles of GR and its W222L mutant

Kinetic differences in the photocycles of WT GR and W222L may also explain the apparently increased light sensitivity of the mutant GR observed under continuous illumination. To illustrate this, let us consider a simplified photocycle scheme, keeping only the ground state (GR) and the rate-limiting O-intermediate, accumulating to the largest extent under such circumstances (Fig. S5†).

Under continuous green-light illumination (and in the absence of red light), the equilibrium concentration of the ground state of GR and O-intermediate can be expressed as follows:
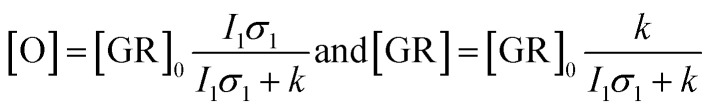
where *I*_1_ denotes the continuous green light intensity, *σ*_1_ includes the absorption cross-sections and the quantum efficiencies of the light-induced photocycle reactions, and *k* stands for the quasi-first order rate constant of the rate limiting thermal back-reaction. The relatively fast, consecutive thermal reactions of the early steps of the photocycle are also included in the *I*_1_*σ*_1_ rate constant.

Hence, assuming a constant green-light exciting intensity, the equilibrium concentration of the O-intermediate is a monotonously growing (while GR is a monotonously descending) function of *k*^−1^, *i.e.*, the elongated O-decay implies an enhanced accumulation of this intermediate under stationary illumination of the W222L mutant, compared to the WT GR.

On similar grounds the accelerating effect of the continuous red measuring light on the O-decay of W222 can be explained if a light-induced short-cut route is assumed in the photocycle (Fig. S6†). In this case, the effective rate constant of the O-intermediate decay becomes *k* + *I*_2_*σ*_2_, where *I*_2_ and *σ*_2_ now correspond to the red light. While, however, for WT GR the effect of red light is negligible (*k* ≫ *I*_2_*σ*_2_), it becomes dominating in the W222 photocycle experiments (*I*_2_*σ*_2_ > *k*). Consistent with our proposal for W222L GR, the O-intermediate to ground state transition is reported^39^ to be slower in W222F GR as well, compared to its WT counterpart.

## Conclusion

In microbial rhodopsins, the all-*trans* retinal chromophore covalently bound through a protonated Schiff base linkage (RPSB) is sandwiched between at least two tryptophan residues, which were proposed to stabilize the highly polar retinal excited state through protein–chromophore interactions. In order to shed light on the electrostatic and steric effects of these highly conserved Trp residues on the stability of the highly polar retinal excited state and thereby its photocycle, we have studied the light-response and photocycle of WT GR and its W222L, W222F and W122L mutants. The quantum yield of *trans*–*cis* retinal isomerization (and initiation of a photocycle) is smaller in the W222L and W222F GR mutants than in WT, which may suggest that the Trp residues are crucial for appropriate charge distribution along the retinal polyene thereby affecting the double bond isomerization quantum yield and direct the isomerization to a specific double bond. The recovery of the initial (resting) state of GR from the O-intermediate is substantially decelerated in the W222L mutant. This suggests that either higher electrostatic interaction or steric effects due to the presence of Trp decrease the re-isomerization energy barrier also in the electronic ground state of the retinal. We note that several vertebrate rhodopsins also contain aromatic amino acid residues, for example, Trp265 and Tyr268 in bovine rhodopsin, which interact with the bound-retinal chromophore.^1^ Therefore, the present work calls for future studies on RPSB–protein interactions of other rhodopsins as well.

## Data availability

The conclusion is based on the data provided in the main paper and in the ESI.† Reasonable requests for additional information can be made to the corresponding authors.

## Author contributions

MS, RM, LZ designed the research; RM, ID, AD, GS, JS, WB, KHJ performed the experiments; RM, ID, AD, MS, LZ analysed the data; RM, AD, MS, LZ writing – original draft, review and editing, and the manuscript was approved by all the authors.

## Conflicts of interest

The authors declare no competing financial interest.

## Supplementary Material

SC-014-D3SC02961A-s001
