# Supplementary material for: Impact of protein–chromophore interaction on the retinal excited state and photocycle of Gloeobacter rhodopsin: role of conserved tryptophan residues
Source: Chem Sci. 2023 Sep 6;14(36):9951–8. doi: 10.1039/d3sc02961a (PMC10510653; doi:10.1039/d3sc02961a)
Supplement: SC-014-D3SC02961A-s001 [file SC-014-D3SC02961A-s001.pdf]

Electronic Supplementary Material (ESI) for Chemical Science.
This journal is © The Royal Society of Chemistry 2023

Supporting Information for

Impact of protein-chromophore interaction on the retinal excited state and photocycle of *Gloeobacter* rhodopsin: Role of conserved tryptophan residues

Ramprasad Misra[1a], Ishita Das[1], András Dér[2], Gábor Steinbach[2,3], Jin-gon Shim[4], Wayne Busse[5], Kwang-Hwan Jung[4], László Zimányi[2*], Mordechai Sheves[1*]

[1]*Department of Molecular Chemistry and Materials Science, Weizmann Institute of Science, Rehovot, 76100, Israel*

[2]*Institute of Biophysics, Biological Research Centre, Eötvös Loránd Research Network, Szeged, H-6726, Hungary*

[3]*Cellular Imaging Laboratory, Biological Research Centre, Eötvös Loránd Research Network, Szeged, H-6726, Hungary*

[4]*Department of Life Science and Institute of Biological Interfaces, Sogang University, Seoul, 04107, South Korea*

[5]*Institute for Biology, Experimental Biophysics, Humboldt-Universitat zu Berlin, Berlin 10115, Germany*

Corresponding authors: L. Zimányi (zimanyi.laszlo@brc.hu);

M. Sheves (mudi.sheves@weizmann.ac.il).

[a]Present address: [2]*Institute for Biology, Experimental Biophysics, Humboldt-Universität zu Berlin, Berlin 10115, Germany*

Expression and Purification of GR and its W22L and W122L Mutants

Codon optimized wild-type (WT) *Gloeobacter* rhodopsin (GR) and its W222L, W122L and W222F mutants were expressed in Luria–Bertani (LB) liquid medium as recombinant protein in *Escherichia coli* (*E. coli*) BL21 (DE3) cells. In each case, *E. coli* transformants were grown in the presence of ampicillin (50 μg mL^{-1}) at 37 °C until the absorption intensity at 600 nm ($O.D._{600}$) reaches between 0.8 - 1.0, followed by induction with 0.1% isopropyl β-D-1-thiogalactopyranoside (IPTG) and 10 μM all-*trans* retinal for 6 hours. Pink-colored cells were harvested by centrifugation (7000 rpm) at 4 °C and resuspended with buffer S (50 mM MeS, 300 mM NaCl, 5 mM imidazole, 5 mM $MgCl_2$; pH 6) that also contains 1% DDM (*n*-dodecyl-β-D-maltoside), DNAse, lysozyme and protease inhibitor. The resulting mixture was then stirred overnight in the dark at room temperature and centrifuged at 18000 rpm at 4 °C for 30 min to collect the colored supernatant. The protein was purified using a Ni^{2+} NTA histidine-tagged agarose column. The column with histidine-tagged protein was first washed with buffer W (50 mM MeS, 300 mM NaCl, 50 mM imidazole, 0.06% DDM; pH 6) and then eluted with buffer E (0.06% DDM, 50 mM Tris–HCl, 300 mM NaCl, 50 mM HCl, 150 mM imidazole; pH 7.5). The eluted protein was washed using Amicon Ultra centrifugal filter devices for three times using a 0.02% DDM solution and 100 mM NaCl to remove the excess imidazole. Similar expression protocol was followed for WT GR and its highly light sensitive GR mutants, although the purification steps of the mutants were done at 4 °C in the dark. All the proteins were stored at 4 °C in 0.06% DDM and 300 mM NaCl before use.

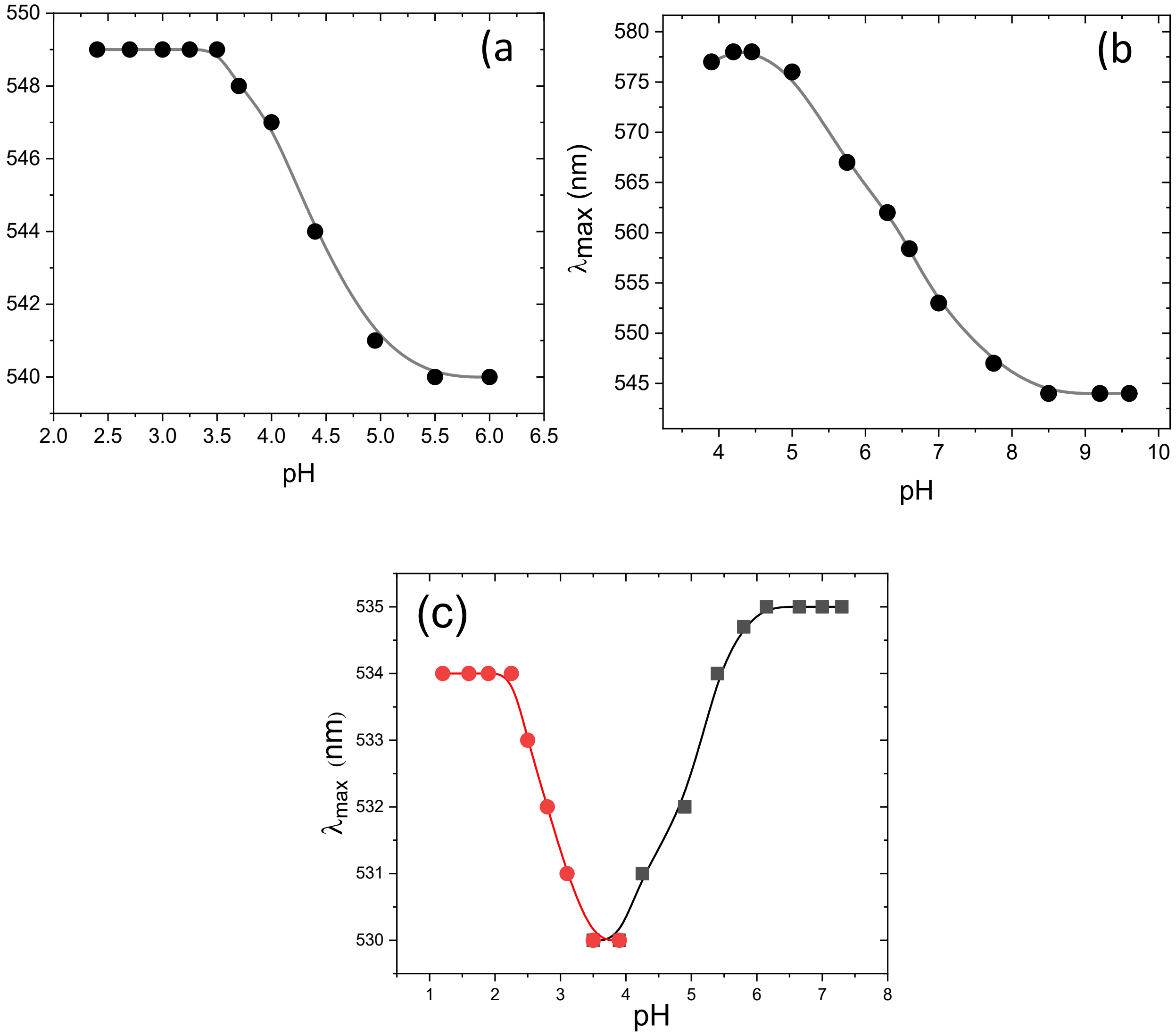

Figure S1: The change of absorption maxima (λ_{max}) of (a) GR and its (b) W222L and (c) W122L mutants due to change of pH. The midpoint of the transition is regarded as pKa of the protonated Schiff base (PSB) counterion.

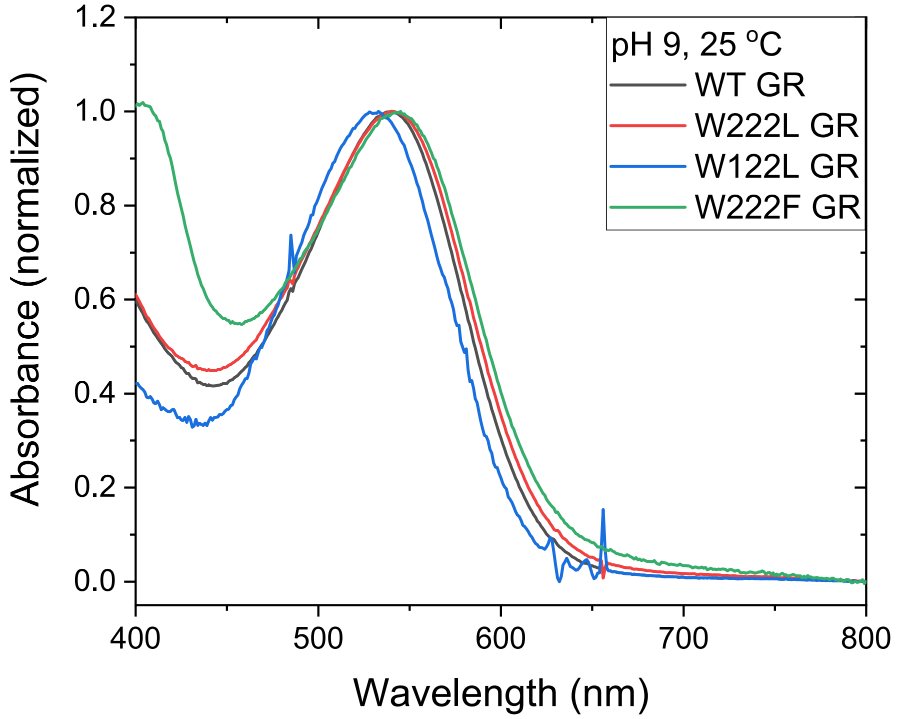

Figure S2: Normalized recorded absorption spectra of WT GR and its W222L, W122L and W222F mutants at 25 °C at pH 9.

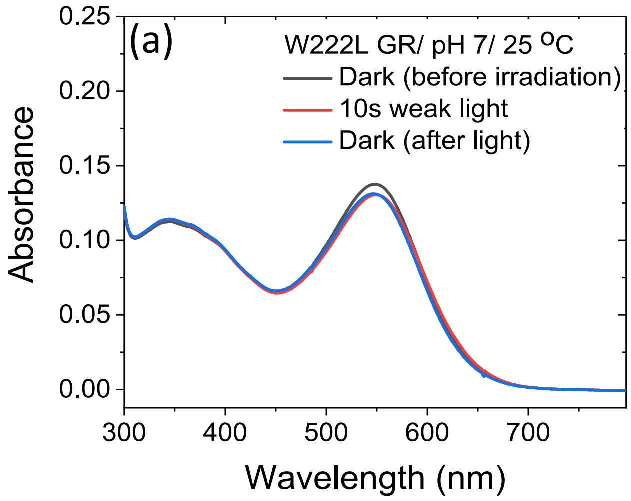

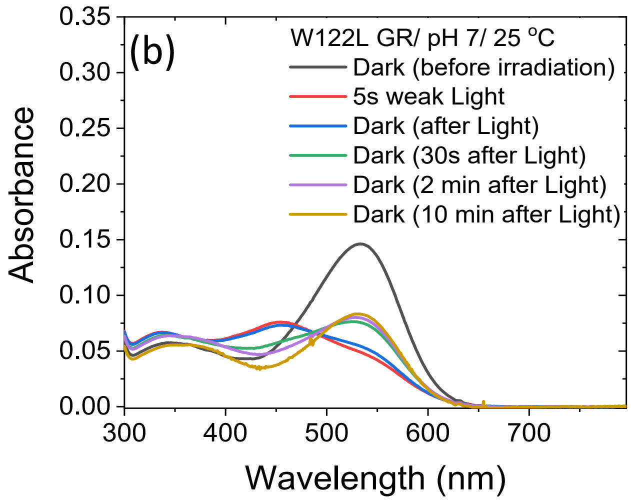

Figure S3: The change in steady-state absorption of (a) W222L and (b) W122L mutants of GR due to irradiation with <500 nm cut-off filter at pH 7. The spectra recorded before, during and after the illumination was terminated are presented.

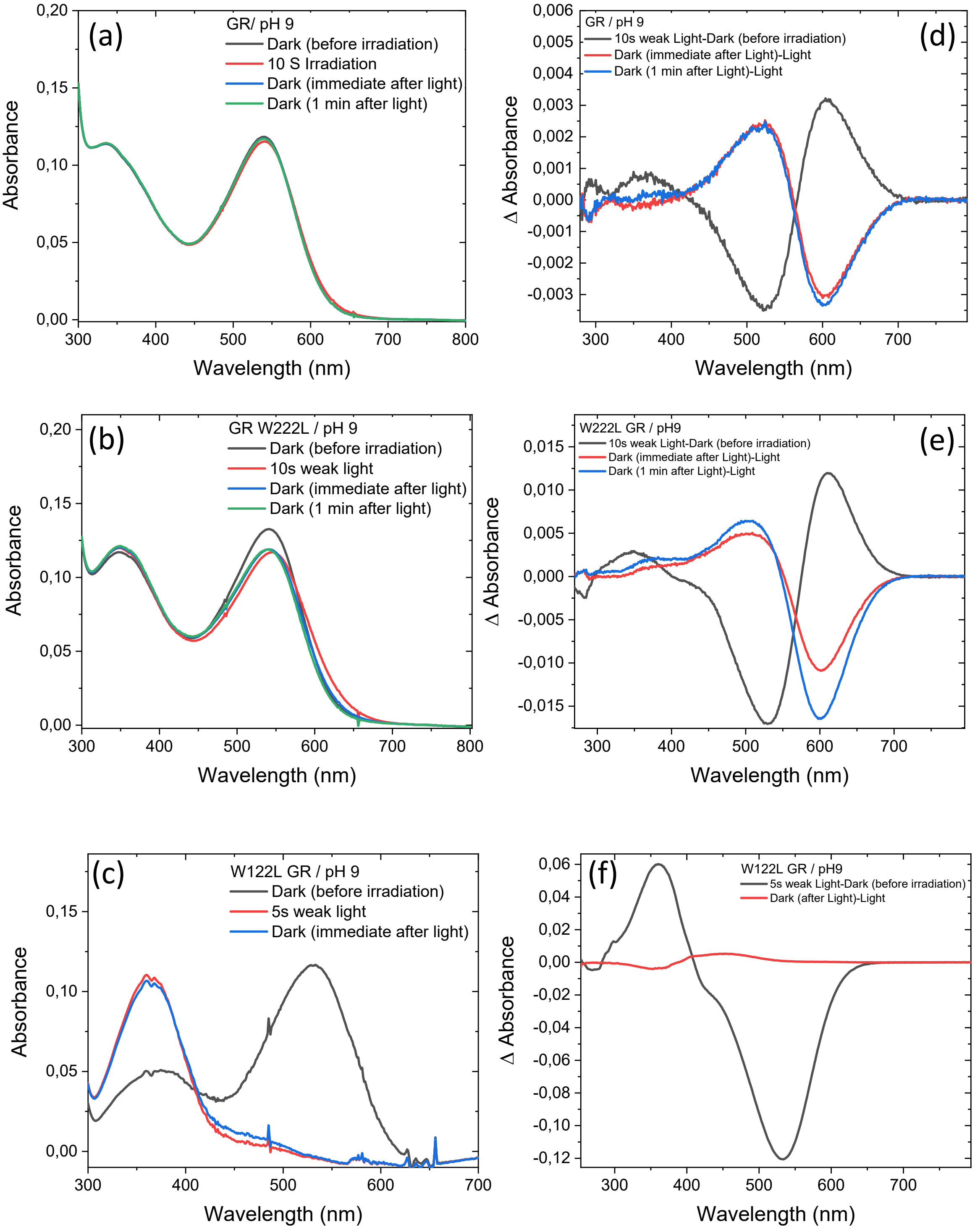

Figure S4: The change in steady-state absorption of (a) GR and its (b) W222L and (c) W122L mutants due to white light irradiation with <500 nm cut-off filter at pH 9. The spectra recorded in the dark after illumination are presented as well. The difference spectra obtained by subtracting the corresponding initial spectra in the dark are presented in panels d-f, respectively.

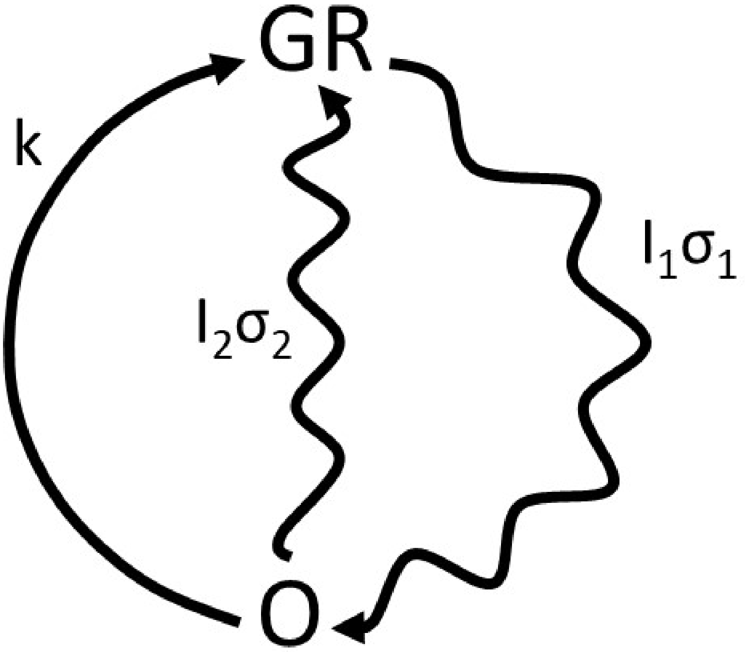

Figure S5: Schematic representation of the rate-limiting O-intermediate in the photocycle of WT GR. The photocycle involving all the intermediates is presented in figure 1.
